# Clinical characteristics and prognosis of acute myocardial infarction in young smokers and non-smokers (≤ 45 years): a systematic review and meta-analysis

**DOI:** 10.18632/oncotarget.21092

**Published:** 2017-09-20

**Authors:** Yuqi Liu, Tianwen Han, Ming Gao, Jinwen Wang, Fang Liu, Shanshan Zhou, Yundai Chen

**Affiliations:** ^1^ Department of Cardiology, PLA General Hospital, Beijing 100853, China; ^2^ ICU of The First Phase Beijing Tsinghua Changgeng Hospital, Beijing 100044, China; ^3^ Department of Cardiology of Anzhen Hospital, Beijing 100029, China

**Keywords:** young, AMI, smoking, prognosis, meta-analysis

## Abstract

The effect of smoking on the prognosis of young patients with acute myocardial infarction (AMI) is inconclusive. We enrolled 2188 young AMI patients (≤ 45 years) from the cardiac center of the Chinese PLA General Hospital and Anzhen Hospital and analyzed their clinical characteristics and prognosis. We also searched the PubMed, EMBASE, and Cochrane Central Register of Controlled Trials electronic databases for January 2001 to March 2017 and considered for inclusion in a meta-analysis those clinical trials that compared prognoses of young smokers and non-smokers with AMI. The proportion of males and alcohol users was higher in young AMI smokers than in non-smokers; the proportion of hypertension was slightly lower. There was no difference in medical treatment between smokers and non-smokers. No differences were evident between smokers and non-smokers regarding in-hospital cardiac events and major adverse cardiovascular events on follow-up, including incidence of stroke. For young AMI patients, smoking did not lead to poorer prognosisin comparison with not smoking. This “smoker's paradox” needs to be confirmed by more randomized controlled multicenter prospective clinical trials.

## INTRODUCTION

Worldwide, the incidence of coronary heart disease (CHD) is increasing. One Chinese report on cardiovascular disease in 2014 estimated that cardiovascular disease globally affected about 290 million people. Accordingly, the burden of cardiovascular disease is rising and has become a major public health problem [1]. With changes in lifestyle, dietary structure, and stress, the age of CHD has been lowered. Acute myocardial infarction (AMI) is a disease with high mortality that presents a serious threat to human life and health. In recent years, the incidence of MI in young people has shown an upward trend. Many epidemiological studies have found that common risk factors of CHD include smoking, male sex, family history of CHD and cerebrovascular disease, dyslipidemia, obesity, hypertension, and diabetes [2–4]. According to the INTERHEART study [5], the risk of smokers developing CHD after quitting smoking shows an annual decrease.

Loukianos et al. determined that persistence of smoking, left ventricular ejection fraction, and reperfusion therapy were independent predictors of major adverse cardiovascular events (MACEs) after adjustment for conventional risk factors [6]. However, other studies arrived at the opposite conclusion: AMI patients who were smokers had survival advantages compared with those who were non-smokers [7–9]. Kang-Yin et al. found that AMI patients who were smokers had lower rates of in-hospital cardiac death and overall mortality than non-smokers [10]. Given the debatable smoking among young patients with AMI in the present study we examined the effect of smoking in young AMI patients on the in-hospital and out of hospital prognosis after the event; we also undertook a systematic review and meta-analysis and compared our findings with those of previous studies.

## RESULTS

### Clinical characteristics of young AMI patients

We evaluated the interaction of smoking with other factors, including gender, alcohol consumption, hypertension, diabetes, hyperlipidemia and family history of CHD. The results showed that interactions with other factors were eliminated (see Supplementary Table 1). The clinical characteristics indicated a higher proportion of males and alcohol consumers among young AMI smokers than in young non-smokers (Table 1; *P* < 0.05). The proportion of hypertension was slightly lower among smokers than in non-smokers. There were no differences in medical treatment (including aspirin, clopidogrel, statin, ticagrelor, ACEIs (angiotensin converting enzyme inhibitors) or ARBs (angiotensin II-receptor-blockers), β-blockers, CCBs (calcium channel blockers), and nitrate) between smokers and non-smokers (Table 2; *P* > 0.05). There was likewise no difference in in-hospital cardiac events and MACEs at follow-up between smokers and non-smokers (Table 3).

**Table 1:** Clinical characteristics of young patients with AMI

Variables	Groups		*P*-value
	Smoker (*n* = 1506)	Non-smoker (*n* = 682)	
Deographic and clinical			
Age (yrs)	39.8 ± 4.7	39.9 ± 4.6	NS
Heart rate (beats/min)	74.2 ± 12.8	73.5 ± 13.2	NS
SBP (mm Hg)	120.8 ± 16.2	120.6 ± 17.0	NS
DBP (mm Hg)	75.7 ± 11.8	76.4 ± 12.7	NS
Body mass index (kg/m2)	28.2 ± 3.2	28.0 ± 3.2	NS
Male (%)	1493 (99.1)	584 (85.6)	0.000
Alcohol use (%)	561 (37.2)	85 (12.4)	0.000
Hypertension (%)	573 (38.0)	302 (44.2)	0.006
Diabetes mellitus (%)	300 (19.9)	118 (17.3)	NS
Hyperlipidemia (%)	475 (31.5)	208 (30.4)	NS
Family historyof CAD (%)	183 (12.1)	72 (10.5)	NS
Prior MI (%)	63 (4.1)	30 (4.3)	NS

**Table 2:** Medical therapy according to the smoker

Variables	Smoker (*n* = 1506)	Non-smoker (*n* = 682)	*P*-value
Aspirin	1427 (94.7)	653 (95.7)	NS
Clopidogrel	1373 (91.1)	630 (92.3)	NS
Statin	1189 (78.9)	571 (83.7)	NS
Ticagrelor	35 (2.3)	23 (3.3)	NS
ACEI/ARB	627 (41.6)	297 (43.5)	NS
ß blocker	1163 (77.2)	527 (77.2)	NS
CCB	150 (9.9)	74 (10.8)	NS
Nitrate	988 (65.6)	452 (66.2)	NS

ACEI: angiotension converting enzyme inhibitors; ARB: angiotensin II receptor blockers; CCB: calcium channel blockers.

**Table 3:** Outcomes in hospital and out hospital according to the smoker

Variables	Smoker (*n* = 1506)	Non-smoker (*n* = 682)	*P*-value
In hospital outcome			
death (%)	19(1.2)	16(2.3)	NS
complications			
Cardiogenic shock (%)	26(1.7)	20(2.9)	NS
Major bleeding (%)	10(0.6)	9(1.3)	NS
AVB (%)	5(0.3)	0	NS
VT or VF (%)	57(3.7)	31(4.5)	NS
Thrombosis (%)	11(0.7)	10(1.4)	NS
CHF (%)	98(6.5)	51(7.5)	NS
Total events (%)	79(5.2)	44(6.4)	NS
Out-hospital outcome	1487	666	
MACE (%)	104(6.9)	49(7.3)	NS
death (%)	2(0.1)	1(0.1)	NS
MI (%)	58(3.9)	36(5.4)	NS
Re-PCI (%)	55(3.6)	31(4.6)	NS
Re-CABG (%)	5(0.3)	0	NS
stroke (%)	57(3.8)	31(4.6)	NS

AKI: acute kidney injury; AVB: atrioventricular block; VT: ventricular tachyarrhythmia; VF: ventricular fibrillation.

The clinical baseline data showed that young AMI patients with in-hospital cardiac events presented a higher heart rate and creatinine kinase (CK), troponin T and glucose levels, and lower red cell count (RCC), platelet count, and ejection fraction than patients without cardiac events (Table 4; *P* > 0.05). The proportion of non-ST-elevation MI was higher in patients with in-hospital cardiac events than in those without such events.

**Table 4:** Clinical characteristics of young patients with AMI

Variables	Groups		*P*-value	Groups		*P*-value
	Non-events in hospital (*n* = 2065)	Events in hospital (*n* = 123)		Non-events out of hospital (*n* = 2098)	Events out of hospital (*n* = 90)	
Deographic and clinical						
Age (yrs)	39.9 ± 4.6	39.4 ± 4.9	NS	39.8 ± 4.7	39.6 ± 4.3	NS
Heart rate (beats/min)	73.4 ± 12.7	79.4 ± 17.7	0.000	73.7 ± 13.0	74.5 ± 14.0	NS
SBP (mm Hg)	120.8 ± 16.3	119.1 ± 19.3	NS	120.7 ± 16.5	120.5 ± 16.2	NS
DBP (mm Hg)	75.9 ± 11.9	75.1 ± 14.3	NS	75.9 ± 12.1	76.7 ± 12.5	NS
BMI (kg/m2)	28.1 ± 3.2	28.3 ± 3.1	NS	28.1 ± 3.1	28.2 ± 3.6	NS
Male (%)	1964(95.1)	113(91.8)	NS	1991(94.8)	86(95.5)	NS
Alcohol use (%)	615(29.7)	31(25.2)	NS	628(29.9)	18(20.0)	0.043
Smoke (%)	1427(69.1)	79(64.2)	NS	1449(69.0)	57(63.3)	NS
Hypertension (%)	816(39.5)	59(47.9)	NS	840(40.0)	35(38.8)	NS
Diabetes mellitus (%)	387(18.7)	31(25.2)	NS	401(19.1)	17(18.8)	NS
Hyperlipidemia (%)	667(32.3)	39(31.7)	NS	653(31.1)	30(33.3)	NS
Family historyof CAD (%)	244(11.8)	11(8.9)	NS	243(11.5)	12(13.3)	NS
Prior MI (%)	86(4.1)	7(5.6)	NS	91(4.3)	2(2)	NS
Biochemical						
TC (mmol/L)	4.73 ± 1.22	4.58 ± 1.07	NS	4.73 ± 1.22	4.61 ± 1.01	NS
TG (mmol/L)	2.75 ± 7.10	2.01 ± 1.29	NS	2.75 ± 7.09	2.00 ± 1.31	NS
LDL-c (mmol/L)	3.15 ± 5.81	2.80 ± 0.88	NS	3.15 ± 5.81	2.83 ± 0.84	NS
HDL-c (mmol/L)	0.96 ± 0.22	0.98 ± 0.26	NS	0.96 ± 0.22	0.99 ± 0.26	NS
Scr (μmol/L)	80.28 ± 38.51	81.15 ± 24.46	NS	80.35 ± 38.51	79.85 ± 23.54	NS
BUN (mmol/L)	7.35 ± 4.82	6.56 ± 4.21	NS	7.34 ± 4.82	6.85 ± 4.34	NS
ALT (U/L)	46.25 ± 35.83	56.76 ± 41.31	NS	46.35 ± 35.88	55.58 ± 41.31	NS
γ-GT (U/L)	51.88 ± 48.94	65.52 ± 83.02	NS	52.44 ± 50.08	66.40 ± 89.18	NS
CK (U/L)	842.38 ± 1286.09	1332.34 ± 1708.09	0.015	848.52 ± 1293.33	1229.67 ± 1640.81	0.049
CK-MB (ng/ml)	79.84 ± 181.77	106.74 ± 148.63	NS	80.29 ± 181.82	98.99 ± 147.31	NS
cTnT (ng/ml)	2.17 ± 2.04	3.25 ± 4.83	0.048	2.20 ± 2.21	2.69 ± 3.31	NS
BNP (pg/ml)	481.87 ± 2659.16	1140.97 ± 3404.97	NS	483.85 ± 2648.81	1199.77 ± 3624.89	NS
Glucose (mmol/L)	6.98 ± 2.87	8.08 ± 4.90	0.048	6.99 ± 2.89	7.94 ± 4.86	NS
Glycosylatedhemoglobin (%)	6.55 ± 1.87	6.10 ± 1.32	NS	6.48 ± 1.82	6.32 ± 1.55	NS
Hematologic						
Hemoglobin (g/L)	147.23 ± 15.54	143.74 ± 16.65	NS	147.22 ± 15.57	143.65 ± 16.09	NS
Red cell count (10*12/L)	5.83 ± 2.04	5.16 ± 1.89	0.003	5.83 ± 2.04	5.21 ± 1.92	0.008
White cell count (10*9/L)	10.42 ± 3.55	10.44 ± 4.27	NS	10.41 ± 3.56	10.53 ± 4.25	NS
PLT (10*9/L)	239.78 ± 61.07	210.60 ± 46.11	0.043	238.03 ± 60.65	215.60 ± 50.72	NS
RDW	12.94 ± 0.71	13.25 ± 0.95	NS	12.94 ± 0.70	13.38 ± 1.03	0.034
Echocardiography						
LVDd (mm)	50.13 ± 5.06	50.15 ± 5.62	NS	50.14 ± 5.13	49.90 ± 4.16	NS
IVST (mm)	10.20 ± 1.58	10.40 ± 1.47	NS	10.22 ± 1.58	10.20 ± 1.29	NS
EF (%)	57.70 ± 8.52	54.53 ± 9.07	0.000	57.45 ± 8.54	58.62 ± 9.48	NS
Diagnosis			0.030			NS
NSTEMI	1648(79.8)	108(87.8)		1685(80.3)	71(78.8)	
STEMI	417(20.1)	15(12.1)		413(19.6)	19(21.1)	

SBP: systolic blood pressure; DBP: diastolic blood pressure; HR: heart rate; BMI: Body Mass Index; NSTEMI: non-ST-segment myocardial infarction; STEMI: ST-segment elevation myocardial infarction; TC: total cholesterol; TG: triglyeride; LDL: low density lipoprotein cholesterol; HDL: high density lipoprotein cholesterol; Cr: creatinine; BUN: blood urea nitrogen; ALT: alanine aminotransferase; AST: Aspartate transaminase; CK: creatine kinase; CKMB: creatine kinase MB isoenzyme; cTnT: Cardiac Troponin T; NT-proBNP: N-terminal pro-B-type natriuretic peptide; HbA1c: hemoglobin A1c; PLT: platelet; RDW: red blood cell distribution width; LVDd: left ventricular end-diastolic dimension; IVST: interventricular septal thickness; EF: ejection fraction; NS: not significant.

With respect to in-hospital cardiac events, alcohol use was lower in patients with MACEs at follow-up than in those without MACEs. At follow-up, the RCC was lower and the CK level and red blood cell distribution width higher in patients with MACEs than in those without MACEs.

### Meta-analysis for prognoses of smokers and non-smokers

#### Prognosis in hospital

**Figure 1:**
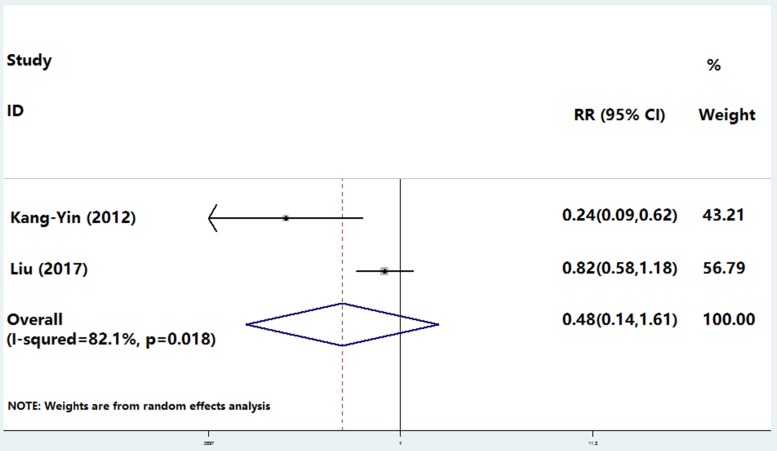
The incidence of in-hospital cardiac events compared between young smokers and non-smokers 3.4% (87/2496) in smokers compared with 5.7% (52/910) in non-smokers (OR, 0.48; 95% CI, 0.14–1.61; *P* 0.235).

Heterogeneity analysis showed *I*^2^ 82.1% and *P* 0.018, and the fixed model was replaced by a randomized model. In two clinical trials, the incidence of in-hospital cardiac events showed no difference between young smokers and non-smokers; the proportion of major cardiac events was 3.4% (87/2496) in smokers compared with 5.7% (52/910) in non-smokers (OR, 0.48; 95% CI, 0.14–1.61; *P* = 0.235; Figure 1). The *P* value of Begg's Test was 0.317.

### Prognosis during follow-up

**Figure 2:**
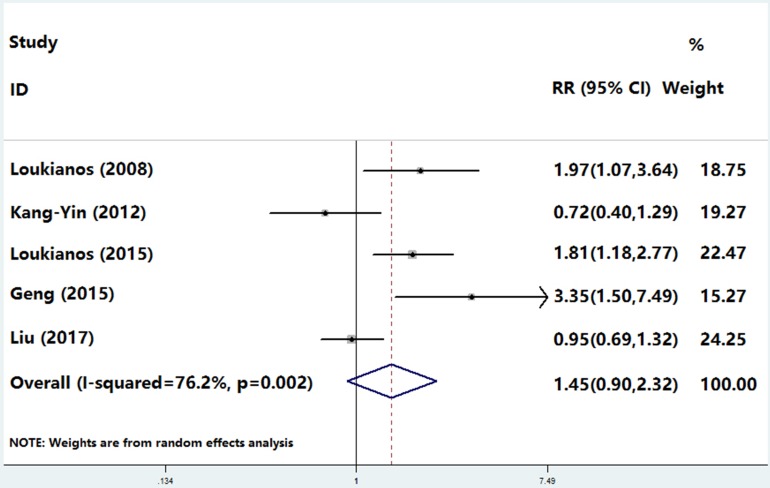
The incidence of MACEs between young smokers and non-smokers 9.8% (270/2755) in smokers compared with 9.0% (103/1140) in non-smokers (OR, 1.45; 95% CI, 0.90–2.32; *P* 0.123)

In five clinical trials, the heterogeneity analysis showed *I*^2^ 76.2% and *P* 0.002, and the fixed model was replaced by a randomized model. During follow-up there was no difference in the incidence of MACEs between young smokers and non-smokers; the proportion of all MACEs was 9.8% (270/2755) in smokers compared with 9.0% (103/1140) in non-smokers (OR, 1.45; 95% CI, 0.90–2.32; *P* = 0.123; Figure 2) with no heterogeneity across the trials (Figure 2 and Supplementary Table 4). The sensitivity analysis showed that small-sample trials (< 500 subjects) showed more cardiac events in the non-smoker group (*P* = 0.001, Supplementary Table 4); however this difference disappeared in larger trials (*P* = 0.434, Supplementary Table 4).

## DISCUSSION

It is accepted by most researchers that smoking is one of the major risk factors of CHD. Smoking may contribute to the occurrence and development of CHD by affecting lipid metabolism, inflammatory reactions, and vascular endothelial dysfunction [14–16]. It is also evident that there is a significant correlation between cigarette smoking and the prognosis of patients with CHD [17, 18]. However, early studies found greater survival among smokers with AMI in comparison with non-smokers; this is termed the “smoker's paradox.” Some researchers believe that this phenomenon may be related to non-smokers being older and having poorer prognosis. Thus, it is necessary to determine the effects of smoking on in-hospital and out-of-hospital prognosis in young AMI patients.

Recently, Kang-Yin et al. found that overall mortality was lower in young smokers with AMI, implying that smoking may have been a protective factor for MI. In the present study, clinical data analysis from our center showed no difference in follow-up between smokers and non-smokers with respect to in-hospital cardiac events and MACEs; one exception was that the incidence of stroke in smokers was lower than in non-smokers. The clinical baseline showed a higher proportion of males and alcohol users and a lower proportion of hypertension among smokers than among non-smokers. There was no difference in medicinal use (aspirin, clopidogrel, statin, ticagrelor, ACEIs/ARBs, β-blockers, CCBs, and nitrate) between the two groups. The meta-analysis found no difference between young smokers and non-smokers with respect to total in-hospital cardiac events. There was likewise no difference between the two groups for total MACEs during follow-up. On further analysis conducted according to the sample size (Supplementary Table 4), surprisingly we found that in the large-sample-trials the good prognosis shown by young smokers in the smaller trials disappeared.

The smoker's paradox is a very important issue, and has led clinicians and patients to make incorrect decisions. The mechanism of this phenomenon warrants further discussion and analysis. Some studies have suggested that it may be caused by the interaction of additional risk factors, such as age [19–21]. However, other studies have found that the phenomenon persisted even after correcting for these possible interference factors. Therefore, researchers have continued to explore the underlying mechanisms of the paradox. Recently it was demonstrated that smoking induces cytochrome P450 1A2 a hepatic enzyme involved in the metabolism of clopidogrel, and causes an increased clopidogrel response among smokers [22]. A subsequent study found that clopidogrel significantly reduced mortality in smokers but not in non-smokers [23]. A more recent study evaluated data from the Clopidogrel in High-Risk Patients with Acute Non-Disabling Cerebrovascular Events (CHANCE) trial [24] and determined that for secondary prevention, smoking status modified the effect of clopidogrel treatment on the outcome [25]. One study showed that clopidogrel likely had an enhanced effect in smokers versus nonsmokers because smoking is an inducer of clopidogrel metabolism, which results in greater degrees of platelet inhibition and less platelet aggregation with clopidogrel [26].

In conclusion, the effect of smoking on MI, especially in young AMI patients, remains questionable. According to our results it could be suggested that other CYP2Y12 receptor antagonists, for example ticagrelor, may be more suitable for young patients with AMI, especially nonsmokers, to bring more clinical benefits. In truth there remains a lack of research on the influence of smoking in the younger population (≤ 45 years) of AMI patients, as only five studies (including the present one from our center) met the criteria of our meta-analysis. In the future, therefore, more randomized controlled multicenter prospective clinical trials with larger sample sizes are needed to further investigate the role of smoking in the prognosis of young AMI patients. In addition, the potential mechanism of smoking in MI, the interaction between smoking and clopidogrel or other antiplatelet drugs, and details of the related mechanisms need to be confirmed by clinical and basic studies.

## MATERIALS AND METHODS

### Subjects

The study protocol has been approved by the Chinese PLA General Hospital and Anzhen Hospital review boards written informed consent and consent for publication has been obtained from all participants. The participants were 2188 patients diagnosed with AMI and ≤ 45 years old who were enrolled from the cardiac center of the Chinese PLA General Hospital and Anzhen Hospital from January 2010 to December 2014. The diagnosis of AMI was made when ST elevation or depression or new left bundle-branch block with chest pain lasted more than 30 minutes and the myocardial enzyme level increased to more than twice the normal range [11]. The exclusion criteria included rheumatic heart disease, cardiomyopathy, congenital heart disease, severe congestive heart disease, malignant tumor, and use of the oral contraceptive pill or pregnancy.

### Clinical database collection and follow-up

All clinical data were derived from hospitalized patients and included sex, age, smoking status, alcohol use, hypertension, hyperlipidemia, diabetes, and family history of coronary artery disease. Laboratory tests included total cholesterol, triglycerides, low-density lipoprotein cholesterol, high-density lipoprotein cholesterol, urea nitrogen, uric acid, creatine kinase MB, troponin T, serum creatinine, N-terminal pro-B-type natriuretic peptide, and routine blood tests. Echocardiographic parameters were assessed using transthoracic echocardiography with the Teichholz method prior to coronary angiography; they included left ventricular ejection fraction, thickness of the interventricular septum and left ventricular end-diastolic inner diameter.

In the hospital, the adverse events recorded included major bleeding, ventricular tachyarrhythmia (VT), ventricular fibrillation (VF), atrioventricular block (AVB), cardiogenic shock and thrombosis. Following hospital discharge, major adverse events were defined as cardiac death, AMI, percutaneous coronary intervention (PCI), coronary artery bypass grafting (CABG), and stroke. All subjects were regularly followed up for 1 year after their first hospitalization.

Complications during hospitalization included cardiogenic shock, AVB requiring temporary cardiac pacemaker insertion, VF or VT requiring anti-arrhythmic drugs or defibrillation and major bleeding. Major bleeding was defined as severe bleeding other than intracranial bleeding. MACEs during the follow-up period included cardiac death, emergency or elective repeat revascularization, AMI, and stroke. Cardiac death was defined as mortality resulting from cardiac disease.

### Literature search

PubMed and Cochrane Central Register of Controlled Trials electronic databases and EMBASE were searched for the period January 2001 to March 2017. Observational or randomized trials that investigated prognoses for smokers and non-smokers among young AMI patients were identified using the following key words: “young,” “AMI,” and “smoking.” All of the studies included in this meta-analysis compared the prognoses for smokers and non-smokers. All studies that met those criteria—regardless of the language or form of publication—were considered eligible for the meta-analysis.

### Selection criteria

We selected only complete, published, non-confounded trials. The inclusion criteria were as follows: (1) randomized controlled clinical trials or observational clinical trials; (2) comparison of prognoses in young smokers and non-smokers (aged ≤ 45 years) with AMI. The exclusion criteria were as follows: (1) clinical outcomes in AMI patients whereby smokers and non-smokers were not reported separately; (2) clinical outcomes of MACEs not reported; and (3) ongoing and duplicated reports or studies. The characteristics of the included studies, including the risk of bias for inclusion with the PEDro scale, are listed in Supplementary Table 2. The clinical characteristics of the clinical studies, if the data were provided in the articles, are listed in Supplementary Table 3.

### Data extraction

**Figure 3:**
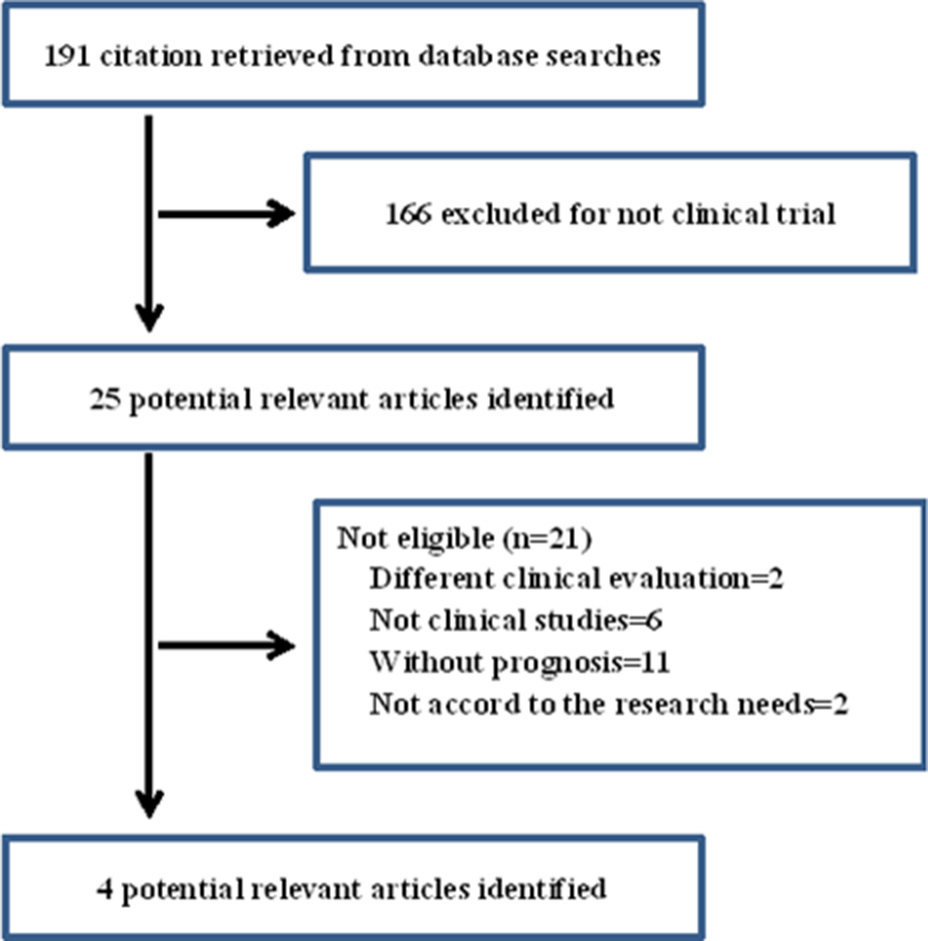
Flow diagram showing the number of citations identified, retrieved, extracted, and included in the final analysis

Two authors (YQ Liu and TW Han) independently performed literature searches to identify all of the trials that met the inclusion criteria. The first authors and year of publication were recorded for each trial. A total of four clinical trials were considered to be potentially relevant (Figure 3).

### Statistical analysis

We performed statistical analyses using SPSS (version 18.0), and Stata software (version 12.0). Continuous variables with normal distributions were expressed as mean ± standard deviation and compared using one-way analysis of variance. Odds ratios (ORs) and 95% confidence intervals (95% CIs) were employed as summary statistics. We calculated ORs for categorical variables using a random-effects model. Heterogeneity between studies was assessed with *I*^2^ statistics [9, 10]. Sensitivity analyses were conducted to assess differences by sample size (< 500 versus ≥ 500) for outcomes. We examined potential publication bias and selection bias using funnel plots and Begg’s, trim-and-fill, and Egger's tests [11]. The reporting of the meta-analysis was performed in compliance with the PRISMA Statement. Four previously reported clinical trials [6, 10, 12, 13] and the results of the present study were included in the meta-analysis. A value of *P* < 0.05 was considered statistically significant.

## SUPPLEMENTARY MATERIALS TABLES


