# Supplementary material for: Clinical characteristics and prognosis of acute myocardial infarction in young smokers and non-smokers (≤ 45 years): a systematic review and meta-analysis
Source: Oncotarget. 2017 Sep 20;8(46):81195–203. doi: 10.18632/oncotarget.21092 (PMC5655274; doi:10.18632/oncotarget.21092)
Supplement: Supplementary file 1 [file oncotarget-08-81195-s001.pdf]

Clinical characteristics and prognosis of acute myocardial infarction in young smokers and non-smokers (≤ 45 years): a systematic review and meta-analysis

SUPPLEMENTARY MATERIALS

Supplementary Table 1: The interactions of smoker and other factors

factors	F	*P*
Smoker*gender	2.313	0.128
Smoker*drink	1.015	0.314
Smoker*hypertension	0.077	0.782
Smoker*diabetes	0.084	0.772
Smoker*hyperlipidemia	0.358	0.550
Smoker*CHD family history	0.180	0.672

Supplementary Table 2: Characteristics of included studies

Studies	Year	Country	Centers	Patients		Follow-up		Type of study	PEDro scale[a]
				smoker	Non-smoker	smoker	Non-smoker		
Geng	2015	Chinese	1	64	88	50 mon	50 mon	Single center, registry trial	5
Loukianos	2008	Greece	1	75	60	10 years	10 years	Single center, registry trial	6
Kang-Yin[a]	2012	Korea	41	990	228	In hospital	In hospital	Multiple center, registry trial	6
Kang-Yin[b]	2012	Korea	41	990	228	8 mon	8 mon	Multiple center, registry trial	6
Loukianos	2015	Greece	2	139	98	9.1 years	9.1 years	Multiple center, registry trial	6
liu[a]	2017	Chinese	2	1506	682	In hospital	In hospital	Multiple center, prospective trial	6
Liu[b]	2017	Chinese	2	1506	682	12 mon	12 mon	Multiple center, prospective trial	6

a: Beaton D, Bombardier C, Guillemin F, Ferraz MB (2002). Recommendations for the Cross-Cultural Adaptation of the DASH & QuickDASH Outcome Measures; Institute for Work & Health; Revised June 2007.

Supplementary Table 3: Clinical characteristics of clinical studies

Studies	Male (%)		Mean age (years)		Hypertension (%)		Hyperlipidemia (%)		DM(%)		PCI(%)	
	smoker	Non-smoker	smoker	Non-smoker	smoker	Non-smoker	smoker	Non-smoker	smoker	Non-smoker	smoker	Non-smoker
Kang-Yin	968	165	40.38 ± 4.68	40.00 ± 6.13	273 (27.6)	78 (34.2)	124 (12.5)	28 (12.3)	147 (14.8)	39 (17.1)	854 (86.3)	175 (77.1)
Liu	1493 (99.1)	584 (85.6)	39.8 ± 4.7	39.9 ± 4.6	573 (38.0)	302 (44.2)	475 (31.5)	208 (30.4)	300 (19.9)	118 (17.3)	1101 (73.1)	509 (74.6)

Supplementary Table 4: Summary estimates for outcomes of young smoker and non-smoker with coronary heart disease

Outcome	*N*	Test of association				Publication bias	
		RR (95%CI)	*Z*	*P*-value	Model	Begg's P	Egger's P
Overall							
Major cardiac events	5	1.45(0.90-2.32)	1.54	0.123	R	0.142	0.345
Sample size < 500							
Major cardiac events	3	2.04 (1.48-2.81)	4.38	0.001	R	0.296	0.279
Sample size > = 500							
Major cardiac events	2	0.89 (0.67-1.18)	0.78	0.434	R	0.317	-
